# Oncogenic action of the exosome cofactor RBM7 by stabilization of CDK1 mRNA in breast cancer

**DOI:** 10.1038/s41523-020-00200-w

**Published:** 2020-10-30

**Authors:** Pei-Wen Xi, Xu Zhang, Lei Zhu, Xin-Yuan Dai, Lin Cheng, Yue Hu, Liang Shi, Ji-Fu Wei, Qiang Ding

**Affiliations:** 1 Jiangsu Breast Disease Center, The First Affiliated Hospital with Nanjing Medical University (Jiangsu Province Hospital), 300 Guangzhou Road, 210029 Nanjing, China; 2 Research Division of Clinical Pharmacology, The First Affiliated Hospital with Nanjing Medical University (Jiangsu Province Hospital), 300 Guangzhou Road, 210029 Nanjing, China

**Keywords:** Breast cancer, Cell growth

## Abstract

RNA exosome can target the specific RNAs for their processing/degradation by distinct exosome cofactors. As a key component in exosome cofactors, RNA binding motif protein 7 (RBM7) shows the binding specificity for uridine-rich sequences in mRNAs via its RNA recognition motifs. However, the specific function of RBM7 in human breast cancer remains unclear. In vitro, experiments revealed that knockdown of RBM7 dramatically inhibited breast cancer cell proliferation, while inducing G1 cell cycle arrest; the opposite was true when RBM7 was overexpressed. Meanwhile, experiments in vivo confirmed the oncogenic function of RBM7 in breast cancer. RNA sequencing and the following pathway analysis found that cyclin-dependent kinase1 (CDK1) was one of the main gene regulated by RBM7. Overexpression of RBM7 increased CDK1 expression, while RBM7 knockdown decreased it. RIP assays additionally found that RBM7 bound directly to CDK1 mRNA. It was also showed that RBM7 could directly bind to the AU-rich elements (AREs) in 3′-UTR of CDK1 mRNA, which contributed to the stability of CDK1 mRNA by lengthening its half-life. More importantly, the oncogenic activity reduced by knockdown of RBM7 could be rescued by overexpression of CDK1 both in vitro and in vivo, but mutant CDK1 failed. All the evidences implied RBM7 promoted breast cancer cell proliferation by stabilizing CDK1 mRNA via binding to AREs in its 3′-UTR. As we knew, it was the first attempt to connect the RNA exosome to the tumor development, providing new insights into the mechanisms of RNA exosome-linked diseases.

## Introduction

Breast cancer is the most common form of cancer affected females^1,2^. The number of new breast cancer cases diagnosed in 2018 was estimated to be 2.1 million (11.6% of all cancer cases)^1^. In addition, it is the 5th leading cause of total cancer mortality for both sexes (9.6 million deaths)^3,4^. So, the detailed molecular mechanism for the breast cancer progression needs further investigation.

Cancer is typically thought to be caused due to mutations in the genome and/or dysregulation at the transcriptional or epigenetic level^5^. Now, the post-transcriptional regulation of mRNAs encoding oncogenic proteins can be co-opted to drive cancer cell phenotypes attracts more attention^6^. The RNA exosome is a ribonuclease complex that is highly conserved between species, playing an essential role in RNA processing and degradation^7,8^. It can target the specific RNAs for their processing/degradation by distinct exosome cofactors. The nuclear exosome targeting (NEXT) complex (hMTR4/SKIV2L2-ZCCHC8-RBM7) has been identified as a key nuclear exosome cofactor in human cells^9–12^. As a key component in NEXT, RNA binding motif protein 7 (RBM7) is an RNA binding protein (RBP), showing the binding specificity for uridine-rich sequences in mRNAs via its RNA recognition motifs (RRMs) motif^13^.

RBPs can interact with mRNAs to control the life cycle of mRNAs, involving all aspects of RNA processing, such as RNA localization, polyadenylation, splicing, transport, stability and translation, and playing an important role in mediating post-transcriptional gene expression^14–16^. Several different RBPs exhibit dysregulation in a range of cancer types, playing roles in altering the proliferation, survival, immune evasion, metastasis, and angiogenesis^17,18^. In breast cancer, we found RBM38 acted as a tumor suppressor by regulating the mRNAs stability of PTEN and c-Myc^19–21^. It was also linked with breast cancer epithelial-to-mesenchymal transition induced by TGF-β via stabilizing zonula occludens-1 mRNA^19^. We also identified another RBP, RBMS2, to stabilize the mRNA of P21 in breast cancer and thereby enhance its regulation^22^. However, the role of RBM7 in cancers, especially in breast cancer, is still unknown.

Herein, we attempted to clarify the biological function of RBM7 in breast cancer, and investigated its potential molecular mechanism by identifying its key mRNA targets. We determined that 1. RBM7 was upregulated in breast cancer and associated with poor survival. 2. RBM7 promoted breast cancer cell proliferation by stabilizing cyclin-dependent kinase1 (CDK1) mRNAs via binding to AREs in its 3′-untranslated region (UTR).

## Results

### RBM7 expression was elevated in breast cancer cells and tissues, related to the poorer prognosis

**Fig. 1:**
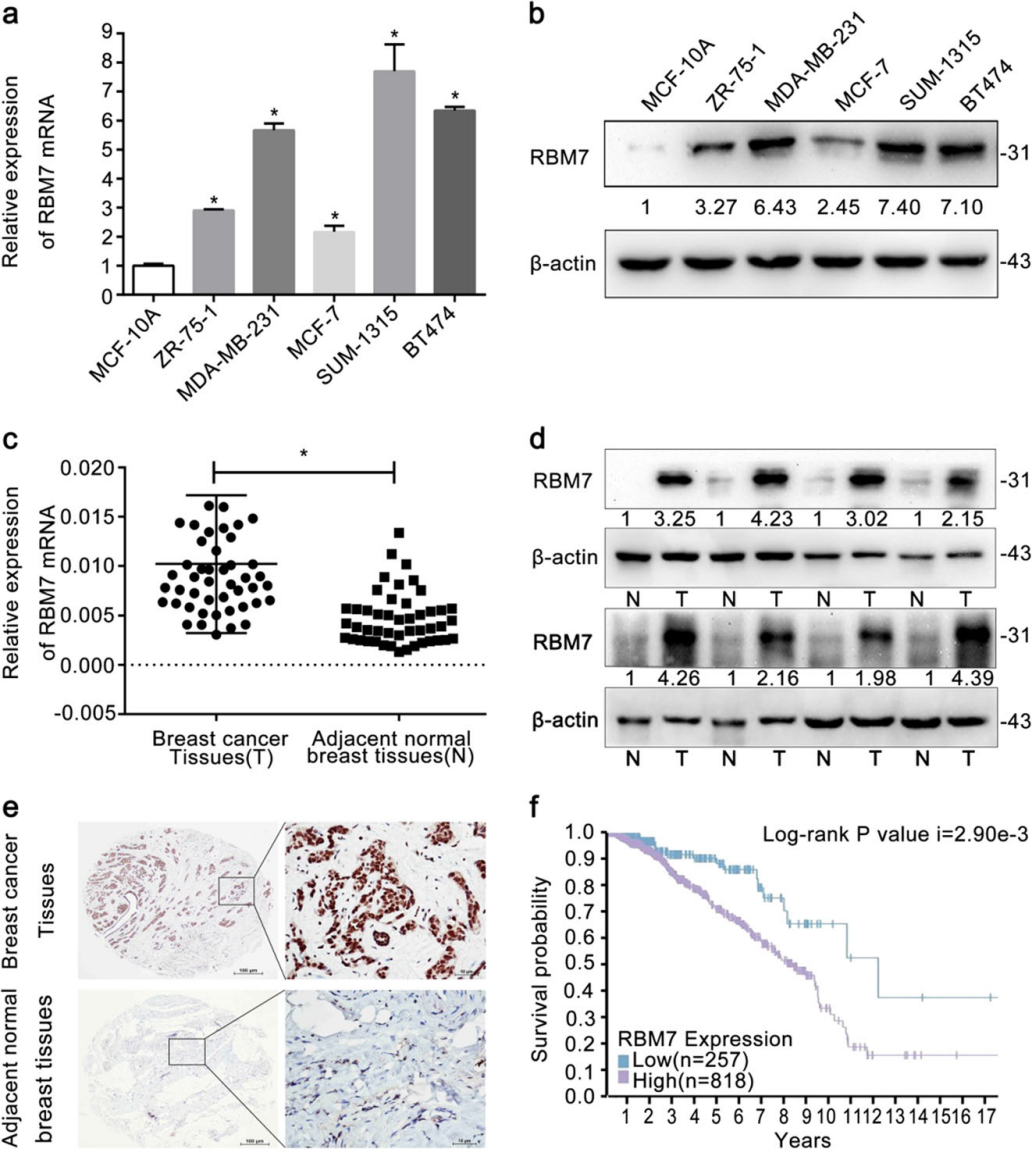
Expression of RBM7 in human breast cancer. **a** RT-qPCR analysis of RBM7 mRNA expression in breast cancer cells and normal epithelial cell line MCF-10A. The relative quantification was calculated by the 2^–ΔΔCt^ method and normalized based on β-actin (*P < 0.05). **b** Western blot analysis of RBM7 expression in breast cancer cells and normal epithelial cell line MCF-10A. The fold change of RBM7 protein was shown below each lane. The intensity of the bands was determined using densitometric analysis. **c** Average expression level of RBM7 mRNA in 46 pairs of human breast cancer tissues and adjacent normal breast tissues. The relative quantification was calculated by the 2^–ΔCt^ method and normalized based on β-actin (*P < 0.05). **d** Expression of the RBM7 protein in eight pairs of breast cancer and adjacent tissues. The fold change in elevated RBM7 was shown below each lane compared to adjacent normal breast tissues. The intensity of the bands was determined using densitometric analysis. **e** Representative images of RBM7 in breast cancer and adjacent normal breast tissues by IHC analysis. RBM7 was mainly expressed in nucleus. Scale bars indicated 100 μm. **f** Survival probability showed that patients with low RBM7 expression (*n* = 257) in breast cancer patients had a better prognosis than those with high RBM7 expression (*n* = 818) group (Log-rank *P* value *i* = 2.90e−3).

RBM7 expression in breast cancer cells including SUM-1315, MDA-MB-231, MCF-7, ZR-75-1, and BT474 cells was found to be higher than that in human normal mammary epithelial cell line MCF-10A at mRNA (Fig. 1a, *P < 0.05) and protein levels (Fig. 1b). Similar to the results from the cell lines, the expression of RBM7 in breast cancer tissues was significantly elevated relative to the adjacent normal breast tissues by quantitative real-time PCR (RT-qPCR) (Fig. 1c, *P < 0.05) and Western blot (Fig. 1d). However, RBM7 expression had no significant associations with different molecular subtypes, including Luminal A, Luminal B, HER2 positive, and Triple negative (Supplementary Fig. 1). Finally, IHC analysis found that RBM7 was mainly expressed in the nucleus in breast tissues (Fig. 1e). The survival curve data of RBM7 from the Human Protein Atlas revealed that upregulation of RBM7 was correlated with poorer prognosis (Log-rank *P*-value *i* = 2.90e−3 < 0.05) in breast cancer patients (Fig. 1f).

### RBM7 promoted breast cancer cell proliferation and growth in vitro

**Fig. 2:**
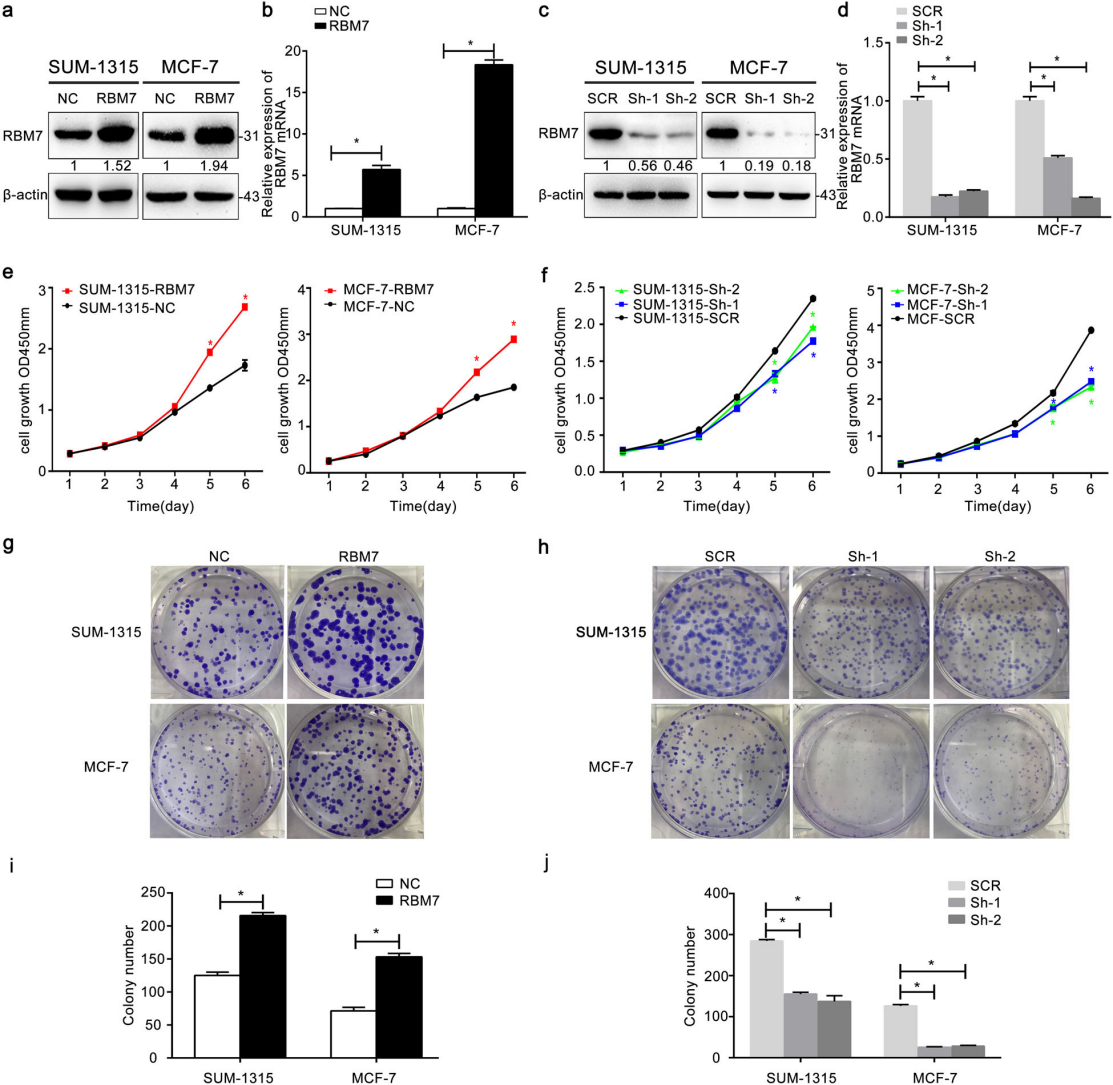
RBM7 promoted the proliferation of breast cancer cells. Western blot was used to demonstrate the efficiency of RBM7 overexpression (**a**) and knockdown (**c**) in SUM-1315 and MCF-7 cells. A fold change in the RBM7 protein was shown below each lane. The intensity of the bands was determined using densitometric analysis. The RT-qPCR was used to verify the efficiency of RBM7 overexpression (**b**) and knockdown (**d**) in mRNA level in SUM-1315 and MCF-7 cells. The relative quantification was calculated by the 2^−ΔΔCt^ method and normalized based on β-actin. The data were represented as mean ± SD (*P < 0.05). The growth of SUM-1315 and MCF-7 cells after overexpression (**e**) or knockdown (**f**) of RBM7 over 6 days was measured using CCK-8 assays. The data were represented as mean ± SD (*P < 0.05). RBM7 indicated RBM7 overexpression in SUM-1315 and MCF-7 cells; NC indicated SUM-1315 and MCF-7 cells transduced with a NC-LV5 negative control vector. Sh-1 and Sh-2 indicated RBM7 knockdown in SUM-1315 and MCF-7 cells; SCR indicated SUM-1315 and MCF-7 cells transduced with a vector-expressing GFP. The growth of cells over 14 days after overexpression (**g**, **i**) and knockdown (**h**, **j**) of RBM7 was measured using colony formation assays. Columns: average of three independent experiments (*P < 0.05). Colonies > 50 mm were counted.

**Fig. 3:**
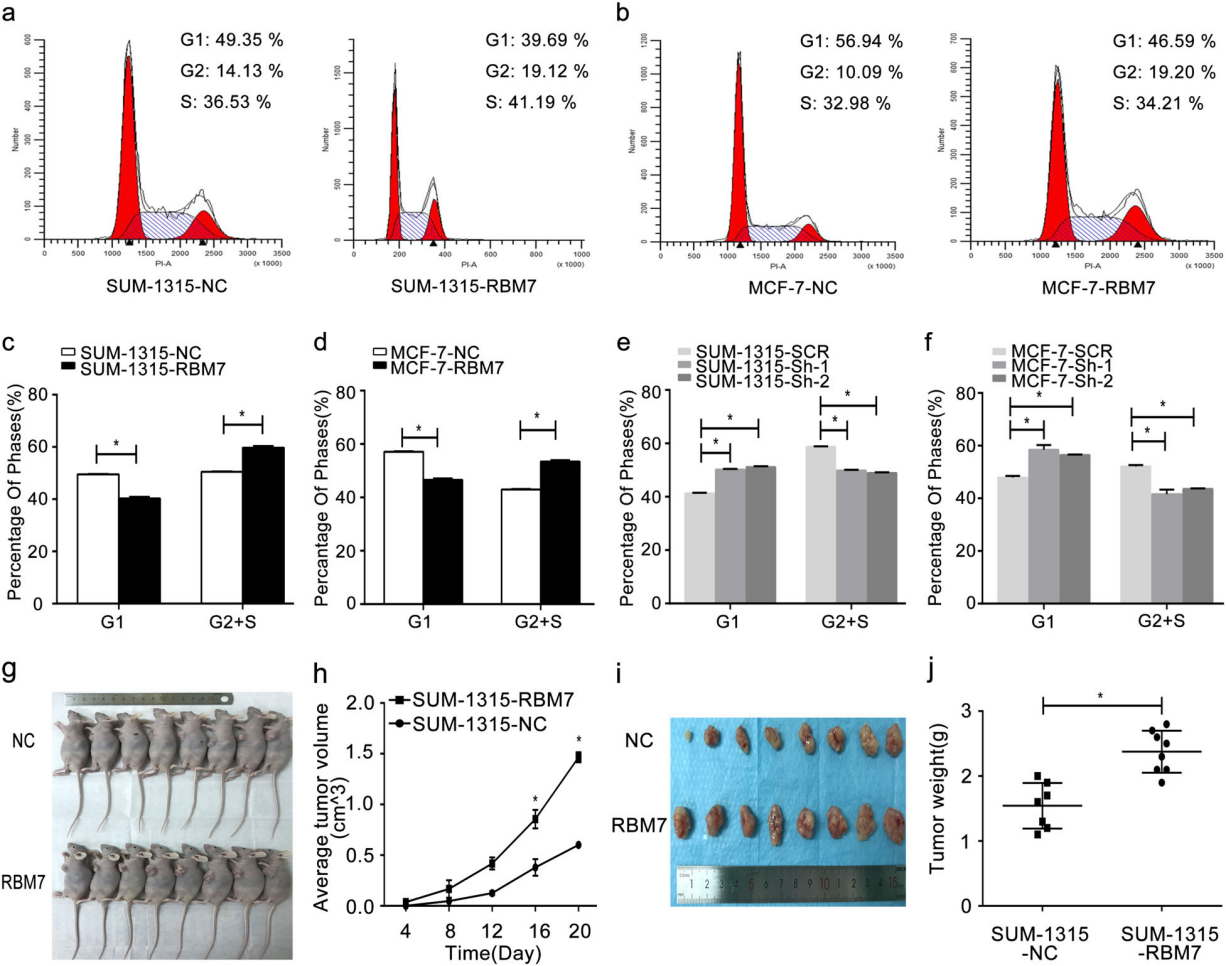
RBM7 promoted breast cancer cell cycle progression in vitro and tumorgenesis in vivo. Cell cycle progression was measured using flow cytometry after overexpression of RBM7 in SUM-1315 (**a**, **c**) and MCF-7 cells (**b**, **d**). Cell cycle progression was also measured after knockdown of RBM7 in SUM-1315 (**e**) and MCF-7 cells (**f**). The data were represented as mean ± SD (*P < 0.05). SUM-1315 cells were injected subcutaneously into the left breast of nude mice (**g**). The tumor volumes growth curves were monitored and recorded every 4 days (**h**). The RBM7 overexpression cells (RBM7) tumor weight was significantly higher, compared to the control cells (NC) (**i**, **j**). Data were represented as mean ± SD (*P < 0.05).

By constructing stably transduced cell lines for 2 weeks, we selected and validated RBM7 expression in MCF-7 and SUM-1315 cells via Western blot (Fig. 2a, c) and RT-qPCR (Fig. 2b, d, *P < 0.05). Owing to Sh-1, Sh-2 showed the efficiencies ≥85% of RBM7 knockdown in breast cancer cells, they were selected for the further study. The CCK-8 assays and the colony formation assays were carried out to examine, whether RBM7 could increase the proliferation of breast cancer cells. For CCK-8 assays, the number of SUM-1315 and MCF-7 cells enhanced from 1.5 to 2.5 fold by overexpressing RBM7 (Fig. 2e, *P < 0.05). In contrast, knockdown of RBM7 significantly dropped the cell growth from 2.5 to 1.5 fold (Fig. 2f, *P < 0.05). Besides, when RBM7 was overexpressed, the SUM-1315 or MCF-7 cells were highly capable of forming colonies (Fig. 2g, i, *P < 0.05). In contrast, cell colony forming ability was significantly reduced, when RBM7 was knockdown (Fig. 2h, j, *P < 0.05). More importantly, CCK-8 and colony formation assays were both performed under the same condition of NC and SCR groups. The results indicated that there was no difference between two groups (Supplementary Fig. 2g–k). To further investigate the cellular function of RBM7 in vitro, we carried out flow cytometry to investigate how RBM7 affected the cell cycle progression. The cell cycle progression of SUM-1315-RBM7 cells was relatively accelerated, compared to SUM-1315-NC cells. Specifically, the number of cells in the G2 and S phases was relatively larger, and the number of cells in the G1 phase was relatively smaller (Fig. 3a). The similar data were observed in MCF-7 cells (Fig. 3b). This meant that the overexpression of RBM7 could cause the proliferation and growth of the breast cancer cells by affecting he process of cell cycle (Fig. 3c, d, *P < 0.05). When RBM7 was knockdown, the similar results were obtained in SUM-1315 cells (Fig. 3e, *P < 0.05) and MCF-7 cells (Fig. 3f, *P < 0.05). The rest of the data were observed in Supplementary Fig. 3 (*P < 0.05). The cell cycle analysis was also conducted under the same condition of NC and SCR groups. The results indicated that there was no difference between two groups (Supplementary Fig. 2h, j).

### RBM7 increased the growth of breast cancer in vivo

To explore the enhanced proliferation and growth capacity of RBM7 in vivo, the cell proliferative capacity of RBM7 was evaluated in SUM-1315 cell in female BALB/C nude mice of 4–6 weeks in size. The time required for tumor formation in the RBM7 overexpression group was significantly shorter than that in the control group (Fig. 3h, *P < 0.05). The tumor was found obviously in control group after 8 days, and the tumors from the RBM7 overexpression group were found less than 8 days. Moreover, RBM7 overexpression group formed larger and heavier tumors, compared to the control group (Fig. 3g–j, *P < 0.05). These data strongly proved that RBM7 could promote breast cancer proliferation in vivo.

### RNA sequencing analysis

**Fig. 4:**
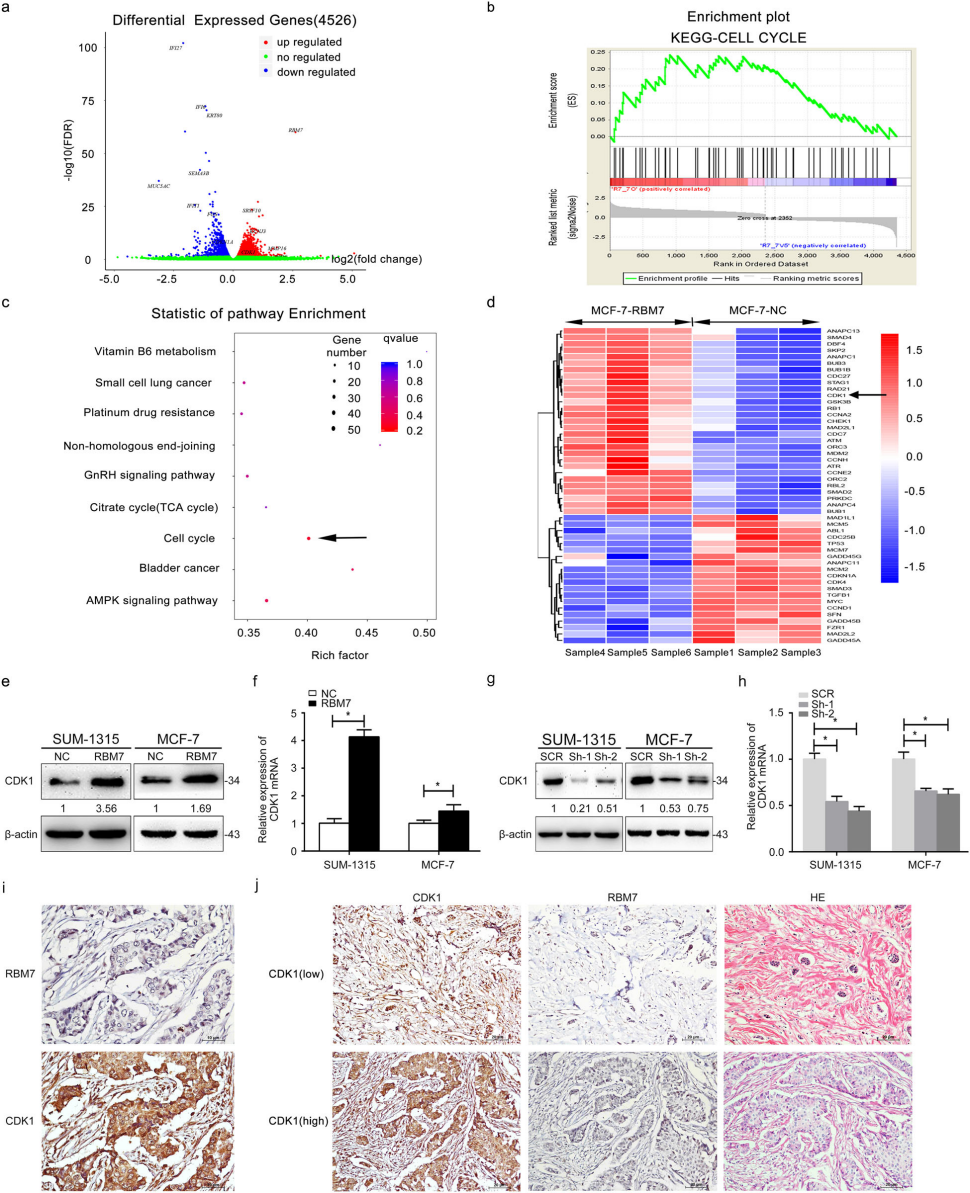
RBM7 regulated CDK1 expression positively. **a** Total 4526 genes were differentially identified from mRNA sequencing. Among them, 2470 genes (red) were upregulated and 2056 genes (blue) were downregulated. **b** Gene set enrichment analysis (GSEA) of differentially expressed genes. **c** Enrichment analysis of the KEGG pathway was used to analyze the distribution of differentially expressed genes in cell cycle pathways. The size and color of the dots represented the number of enriched genes and *q* values, respectively. **d** Heatmap represented the downregulated and upregulated genes measured in MCF-7 cells. The expressions of CDK1 and RBM7 were marked. The arrow indicated that CDK1 was upregulated in the RBM7 overexpression group. **e**–**h** The overexpression of RBM7 significantly increased CDK1 (**e**, **f**), whereas the knockdown of RBM7 deceased the expression of CDK1 in SUM-1315 cells. Similar results were observed in MCF-7 cells (**g**, **h**). The intensity of the bands was determined using densitometric analysis. The fold changes were shown below each lane. The relative quantification was calculated by the 2^−ΔΔCt^ method and normalized based on β-actin (*P < 0.05). **i** RBM7 expression was positively related to CDK1 in breast cancer tissues. The immunohistochemistry analysis of CDK1 and RBM7 in breast cancer tissue was at 400× magnification. The arrow showed the location of RBM7 and CDK1 was mainly expressed in nucleus. Scale bars indicated 10 μm. **j** Representative images of moderate and the relative RBM7 staining in CDK1 high and low positive breast cancer tissues (*n* = 64, *P < 0.05) by IHC staining. Scale bars indicated 20 μm. The correlation between RBM7 expression and clinic pathological features was showed in Table 1 (*P < 0.05).

The RBM7 overexpression and control group in MCF-7 cells were used to find the possibly affected genes, and downstream pathways of RBM7 by RNA sequencing analysis. First, it was realized that 4526 genes were differentially expressed, of which 2470 genes were upregulated and 2056 genes were downregulated (Fig. 4a). Next, gene set enrichment analysis (GSEA) revealed that significantly expressed genes affected by RBM7 were mainly concentrated in the cell cycle (Fig. 4b, c). Furthermore, GO enrichment analysis of the DEGs was implemented to find that CDK1 was the mainly upregulated gene in the downstream of RBM7 (Fig. 4d).

**Table 1:** Association of RBM7 with CDK1 and clinic pathological characteristic of breast cancer.

Clinic pathological characteristic	RBM7 expression			
	No. of cases	Low	High	*P*-value
CDK1				0.000002
Low	16	11	5	
High	61	6	55	
Age (years)				0.445065
≥50	39	10	29	
<50	38	7	31	
Tumor size (cm)				0.227126
≤2	31	9	22	
>2	46	8	38	
TNM stage				0.413091
I–II	26	6	20	
III–IV	6	3	3	
Lymph node metastasis				0.647612
Absent	40	8	32	
Present	37	9	28	
Pathological grade				0.838464
I–II	9	3	6	
III	7	2	5	

### RBM7 regulated CDK1 expression in breast cancer

A pivotal increase of CDK1 expression was observed in protein (Fig. 4e) and mRNA (Fig. 4f, *P < 0.05) levels when RBM7 overexpression in SUM-1315/MCF-7 cells. Besides, the expression of protein and mRNA of CDK1 in RBM7 knockdown SUM-1315 and MCF-7 cells was significantly decreased (Fig. 4g, h, *P < 0.05). Finally, IHC revealed that CDK1 expression was higher in RBM7 higher expressed breast cancer tissues (Fig. 4i, j, *P < 0.05). This indicated that RBM7 expression had a positive relationship with CDK1 expression in breast cancer tissues. All these implied that RBM7 could significantly affect the expression of CDK1 in breast cancer.

### RBM7 could increase CDK1 mRNA stability

**Fig. 5:**
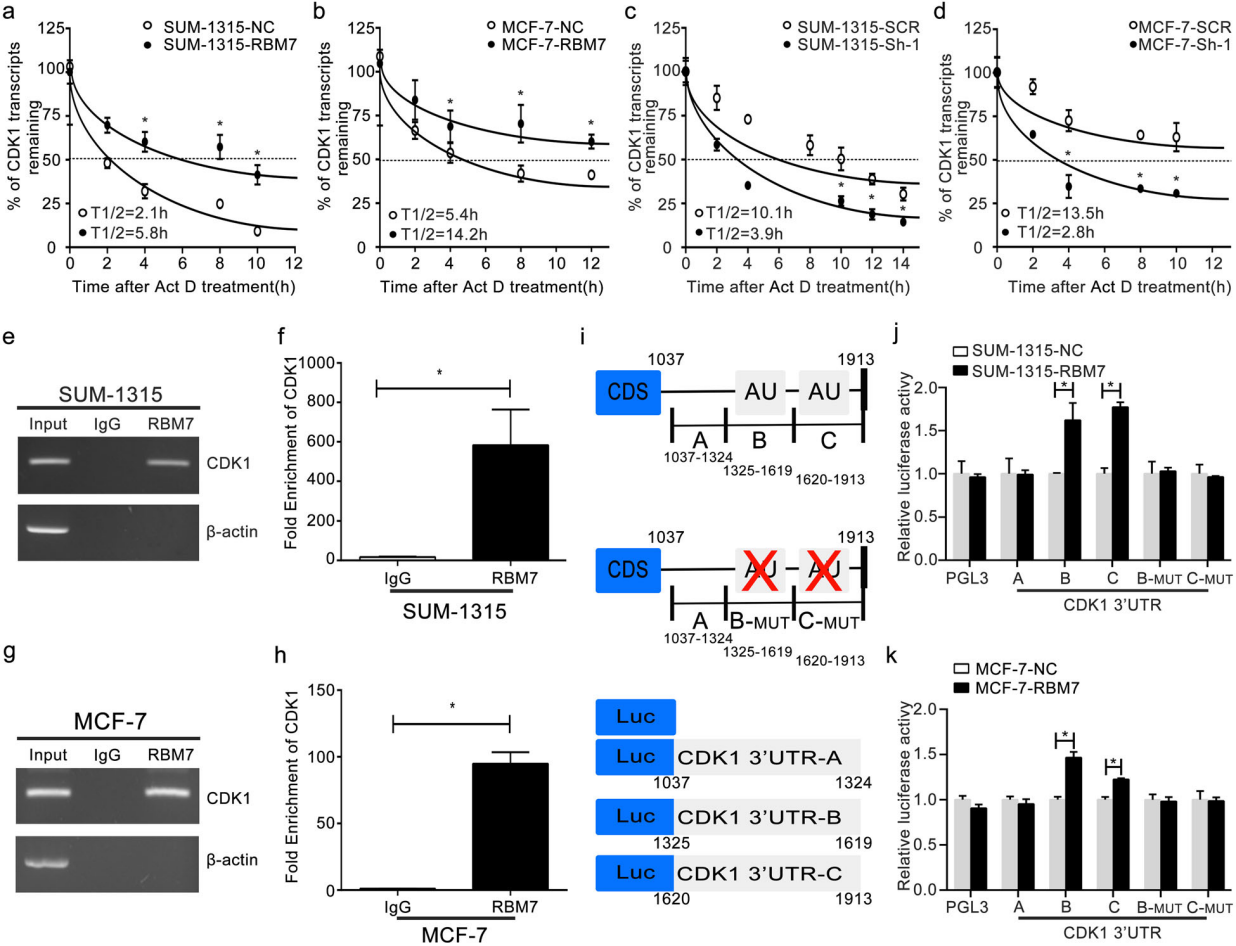
RBM7 directly bound to the 3′-UTR of CDK1 mRNA to stabilize the CDK1 transcript. **a**, **b** The half-life of CDK1 transcript was increased after RBM7 overexpression in SUM-1315 (**a**) and MCF-7 (**b**) cells. The control (NC) and RBM7 overexpression (RBM7) cells were treated with 5 µg/ml Act D for 0, 2, 4, 6, 8, 10, 12, and 14 h. **c**, **d** The half-life of CDK1 transcript was decreased after RBM7 knockdown in SUM-1315 (**c**) and MCF-7 (**d**) cells. The control (SCR) and RBM7 knockdown (Sh-1) cells were treated with 5 µg/ml Act D for 0, 2, 4, 6, 8, 10, 12, and 14 h. The relative quantification was calculated by the 2^−ΔΔCt^ method and normalized based on β-actin. **e**, **f** SUM-1315 and **g**, **h** MCF-7 cell lysates were immunoprecipitated with RBM7 antibody. PCR (**e**, **g**) and RT-qPCR (**f**, **h**) measured transcript levels of CDK1 and β-actin within RBM7 or IgG immunocomplexes in MCF-7 and SUM-1315 cell lysates (*P < 0.05). **i** Schematic diagram of various regions in the 3′-UTR of CDK1 mRNA. **j**, **k** The reporter containing CDK1 3′-UTR-B, C was increased by overexpression of RBM7 in SUM-1315 (**j**) and MCF-7 (**k**) cells. Data were shown as mean ± SD (*P < 0.05).

When actinomycin-D (Act D, 5 µg/ml) was used to treat cells for a range of times (0–14 h), the half-life of the CDK1 mRNA increased from 2.1 to 5.8 h (Fig. 5a, *P < 0.05) in RBM7 overexpression SUM-1315 cells. In RBM7 overexpression MCF-7 cells, the half-life of CDK1 mRNA rose from 5.4 to 14.2 h (Fig. 5b, *P < 0.05). Additionally, the half-life of CDK1 mRNA was reduced after RBM7 knockdown. In SUM-1315 cells, the half-life of CDK1 mRNA dropped from 10.1 h in control cells to 3.9 h in RBM7 knockdown cells (Fig. 5c, *P < 0.05). Similarly, the half-life of CDK1 mRNA fell from a control cell value of 13.5–2.8 h in RBM7 knockdown MCF-7 cells (Fig. 5d, *P < 0.05). All the data indicated that RBM7 could increase CDK1 expression via regulating its mRNA stability. The RNA stability assays of NC and SCR groups were also performed under the same condition. The results indicated that there was no difference between two groups (Supplementary Fig. 2l).

### RBM7 bound to the AU-rich elements (AREs) in 3′-UTR of CDK1 mRNA directly

RIP assays together with RT-PCR (Fig. 5e, g) and RT-qPCR (Fig. 5f, h, *P < 0.05) found that CDK1 mRNA was evident in RBM7 but not in the control IgG immune complex both in SUM-1315 and MCF-7 cells, suggesting that CDK1 mRNA formed an immune complex with RBM7 protein. As a means of confirming whether RBM7 was able to specifically bind the AREs present within the 3′-UTR region of the CDK1 mRNA, luciferase reporter assay was conducted by designing a pGL3 reporter that contained A, B, B-_MUT_, C and C-_MUT_ 3′-UTR regions (Fig. 5i). While AREs were present within the 3′-UTR-B and C constructs, they were absent in 3′-UTR-A. Moreover, 3′-UTR-B-_MUT_ and 3′-UTR-C-_MUT_ were described as the deletion mutation of AREs. Consistent with this, the 3′-UTR-B and C constructs yielded significantly better luciferase activity in RBM7 overexpression of SUM-1315 and MCF-7 cells relative to 3′-UTR-A, B-_MUT_, and C-_MUT_ (Fig. 5j, k, *P < 0.05). This implied that RBM7 could bind to the AREs in 3′-UTR of CDK1 mRNA and increased the expression of CDK1.

### CDK1 rescued a decrease in cell proliferation and growth induced by knockdown of RBM7 both in vitro and in vivo

**Fig. 6:**
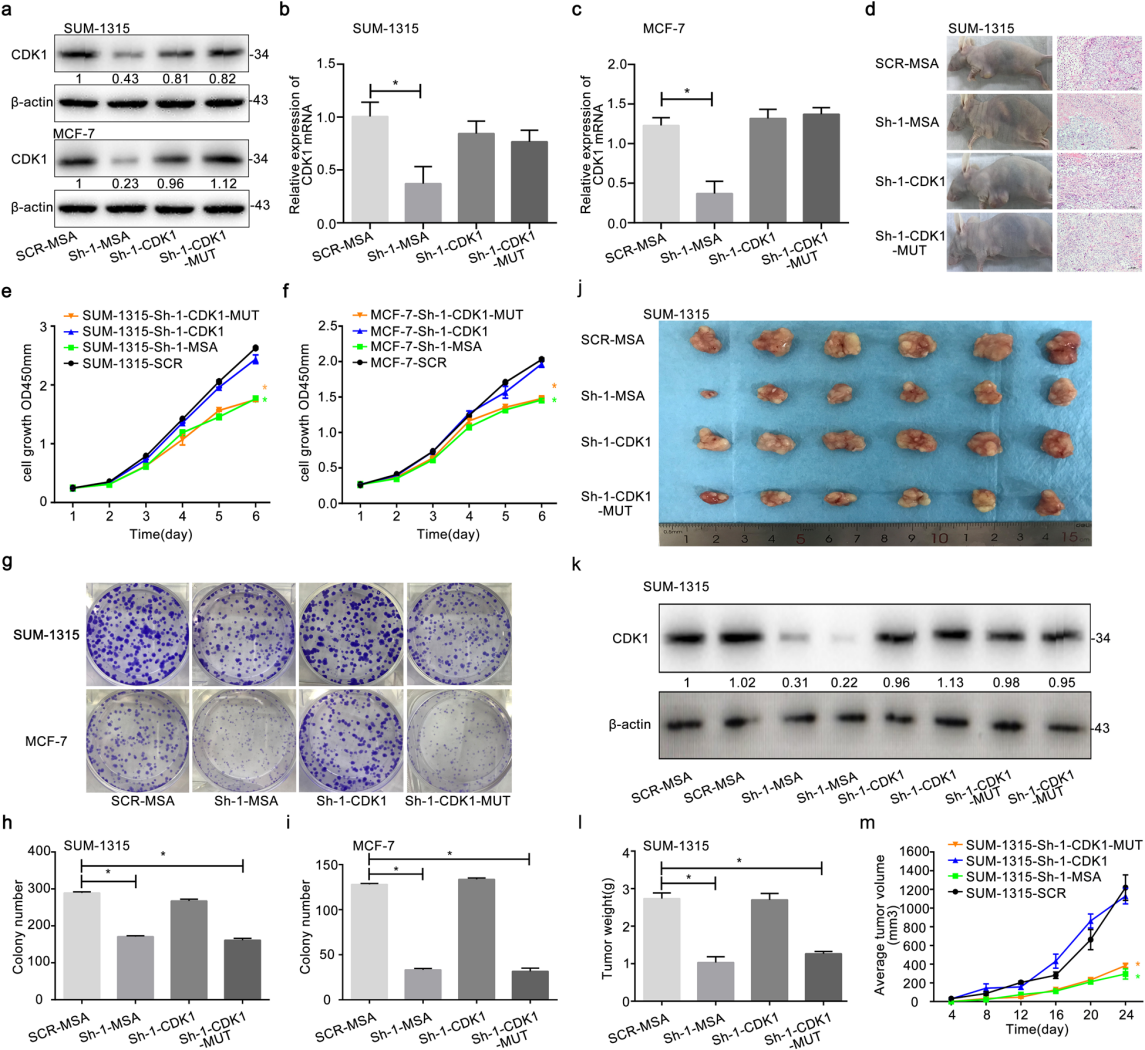
CDK1 rescued a decrease in cell proliferation and growth induced by knockdown of RBM7. **a** Western blot and **b**, **c** RT-qPCR were used to verify the efficiency of CDK1 overexpression and CDK1 mutant overexpression in the knockdown of RBM7 or control in SUM-1315 and MCF-7 cells. The relative quantification was calculated by the 2^−ΔΔCt^ method and normalized based on β-actin (*P < 0.05). The fold change of CDK1 protein was shown below each lane. The intensity of the bands was determined using densitometric analysis. Cell proliferation assays of RBM7 knockdown or control in SUM-1315 (**e**) and MCF-7 (**f**) cells after cotransfection with CDK1 or CDK1 mutant by CCK-8. Data represented as mean ± SD (*P < 0.05). Colony formation assays of RBM7 knockdown or control in SUM-1315 (**h**) and MCF-7 (**i**) cells after co-transfection with CDK1 or CDK1 mutant. Representative photographs (upper) and quantification (lower) were shown (**g**–**i**). SCR-MSA represented the control cells; Sh-1-MSA represented RBM7 knockdown cells; Sh-1-CDK1 represented RBM7 knockdown + CDK1 overexpression cells; Sh-1-CDK1-MUT represented RBM7 knockdown + CDK1-MUT overexpression cells. **d** SUM-1315 cells were injected subcutaneously into the left breast of nude mice. The tumor tissues were stained with H&E. Scale bars indicated 20 μm. **j** The photograph represented the tumor tissues in RBM7 knockdown + CDK1 overexpression (Sh-1-CDK1), RBM7 knockdown + CDK1-MUT overexpression (Sh-1-CDK1-MUT), RBM7 knockdown (Sh-1-MSA) and the control group (SCR-MSA). **k** CDK1 expression of the tumor tissues was elevated by Western blot analysis. The fold change of CDK1 protein was shown below each lane. The intensity of the bands was determined using densitometric analysis. **l** CDK1 reversed the tumor weights decrease induced by RBM7 in vivo. The tumor weights were got on day 24. **m** The tumor volumes growth curves were monitored and recorded every 4 days. The data were represented as mean ± SD (*P < 0.05).

CDK1 and CDK1 mutant overexpression could reverse the reduction of CDK expression by the knockdown of RBM7 both in protein level (Fig. 6a) and mRNAs (Fig. 6b, c, *P < 0.05). Moreover, CDK1 overexpression could reverse the inhibited proliferation and growth of SUM-1315 and MCF-7 cells by knockdown of RBM7 during CCK-8 and colony formation assays. But mutant CDK1 failed (Fig. 6e–i, *P < 0.05). Furthermore, SUM-1315 cells (SCR-MSA, Sh-1-MSA, Sh-1-CDK1, and Sh-1-CDK1-MUT) were respectively injected into the mammary gland of the female nude mice (Fig. 6d). Overexpressing CDK1 significantly reversed the decreased weights and volumes of formed tumors by the knockdown of RBM7, whereas the CDK1-MUT did not (Fig. 6j–m, *P < 0.05). Interestingly, CDK1 expression of tumor tissues in protein levers further showed that the tumor volume and weight indeed correlated with CDK1 expression (Fig. 6k). All these results demonstrated that CDK1 could rescue the weakening of cell proliferation and growth caused by RBM7 knockdown in vitro and in vivo.

## Discussion

RBM7, an RBP in one of RNA exosome cofactors (NEXT complex), was gradually confirmed as a key regulatory role in breast cancer development^23–26^. Moreover, upregulated expression of RBM7 in breast cancer tissues was positively correlated with poor prognosis, implying its potential prognostic value for breast cancer patients.

In vitro, knockdown of RBM7 dramatically inhibited breast cancer cell proliferation, while inducing G1 cell cycle arrest; the opposite was true when the expression of RBM7 was overexpressed^27–30^. Meanwhile, experiments in vivo confirmed the oncogenic function of RBM7 in breast cancer. All these results strongly supported that RBM7 acted as an oncogenic role in breast cancer. It was previously found that two other RBMs, RBM38 and RBMS2 acted as tumor suppressor in breast cancers^19,22^. Yang et al. also found RBMS3 could inhibit breast cancer proliferation and metastasis^31,32^. In the present study, RBM7 firstly served as a breast cancer oncogene. It implied that every RBM needed more detailed investigation related to its role in breast cancer.

Furthermore, RNA sequencing and pathway analyses identified the primary targets of RBM7. It was showed that RBM7 potentially regulated the genes related to cell cycle. Among these genes, CDK1 was found to be one of the main genes regulated by RBM7. Obviously, CDK1 is termed as an essential point to drive all cell cycle stages in mammals, executing key steps in the cell division process^33–35^. Our study confirmed that the downregulation of RBM7 significantly increased the number of cells in G1 and decreased the number of cells in G2 + S phase (and vice versa). Nevertheless, the number of cells in G2 and S phases both decreased, the trend was not as obvious as that in G1 phase. Here, it was our focus that the downregulation of RBM7 caused an arrest in G1 phase and stopped in cell mitosis. Abnormal expression of CDK1 can induce irregular proliferation, genomic, and chromosomal instability of tumors, thus affecting the occurrence and development of tumors^36–38^. More importantly, selective CDK1 inhibition can provide therapeutic benefits for some human tumors. In the present study, overexpression of RBM7 increased CDK1 expression, while RBM7 knockdown decreased it. RBM7 was capable of increasing CDK1 mRNA stability by lengthening its half-life, then RIP assays represented that RBM7 directly bound CDK1 mRNA. It was further enriched that RBM7 could directly bind to the AREs in 3′-UTR of CDK1 mRNA, contributing to CDK1 mRNA stability. More importantly, the oncogenic activity reduced by knockdown of RBM7 could be rescued by overexpression of CDK1 both in vitro and in vivo, but mutant CDK1 failed. It indicated CDK1 was a main target of RBM7 exhibiting its oncogenic activity in breast cancer. All the evidences implied RBM7 promoted breast cancer cell proliferation by stabilizing CDK1 mRNAs via binding to AREs in its 3′-UTR.

RNA exosome can target the specific RNAs for processing/degradation by distinct exosome cofactors^39,40^. How this complex mediates precise processing of some RNA targets and complete destruction of other RNAs is still poorly understood. Moreover, the mutations in these RNA exosome genes have been reported to different tissue-specific diseases^41,42^. Major challenges in understanding the function of the RNA exosome still remain. The abnormal expression of RBM7, described as a key component in nuclear exosome cofactor (NEXT complex), could show the oncogenic activities in breast cancer due to its RBP activities^43^. As we knew, it was the first attempt to connect the RNA exosome to the tumor development, providing new insights into the mechanisms of RNA exosome-linked disease.

## Methods

### Cell lines and cell cultures

The breast cancer cell lines SUM-1315, MCF-7, BT474, ZR-75-1, and MDA-MB-231 (ATCC, USA) were grown in humidified atmosphere containing 5% CO_2_ at about 37 °C. DMEM (Wisent, China) containing glucose (4.5 mg/ml), 10% FBS (Gibco, USA), penicillin (100 μg/ml) and streptomycin (100 μg/ml, Hyclone, USA) was used for cell culture.

### Quantitative real-time PCR (RT-qPCR)

Total RNA from cells was isolated by using Trizol (TaKaRa, Kusatsu, Japan), and cDNAs were synthesized with the Primescript RT Reagent (TaKaRa, Kusatsu, Japan). RT-qPCR was conducted as in previous studies^19^. The PCR procedure for amplification is 94 °C for 30 s, 94 °C for 30 s, 55 °C for 30 s, 72 °C for 1 min, and 72 °C for 10 min. The cycle of related genes was repeated 35 times from the second step to the fourth step. The sequences of primers were listed as follow.

β-actin-F: 5′-TCACCCACACTGTGCCCATCTACG A-3′

β-actin-R: 5′-CAGCGGAACCGCTCATTGCCAATGG-3′

RBM7-F: 5′-GAAGCGGATCGCACTCTCTTT-3′

RBM7-R: 5′-CACAAACGCAAACTGCTTTGG-3′

CDK1-F: 5′-AAACTACAGGTCAAGTGGTAGCC-3′

CDK1-R: 5′-TCCTGCATAAGCACATCCTGA-3′

### Western blot

Extracted proteins were performed as previously described^44^ by using the following primary antibodies: antirabbit RBM7 (Abcam, Cambridge, MA, USA and Sigma, USA), antirabbit CDK1 (Proteintech, USA), antimouse β-actin (Beyotime Biotechnology). Cell Signaling Technology provided all secondary antibodies. Antibodies were diluted based on manufacturer’s recommendations. All the blots derived from the same experiment and were processed in parallel. Full and uncropped images of blots were showed as supplementary figures in the Supplementary Information file. Molecular weight markers were Rainbow markers from Thermo Fisher Scientific Inc (10–180 kDa).

### Lentivirus transduction

SUM-1315 and MCF-7 cells were transduced using appropriate lentiviral constructs (Genepharm, Shanghai, China) to overexpress or knockdown RBM7. Selection to isolate stably tranduced cells was conducted using puromycin (3 μg/ml) over a 2 week period. The breast cancer cell lines of RBM7 were stably transduced with RBM7 overexpression lentivirus (referred to as RBM7) and NC-LV5 negative control vector (referred to as NC). And the breast cancer cell lines of RBM7 knockdown was transduced using a negative control LV3-pGLV-h1-GFP-puro vector (referred to as SCR) and RBM7 knockdown lentivirus (referred to as Sh-1, Sh-2, and Sh-3). For recovery assays, we transduced CDK1 lentivirus (Obio Technology, Shanghai, China) on the basis of cells which had transduced with the knock-down and control lentivirus of RBM7. Zeocin (300 μg/ml) was used over a 2 week period for stably transduced cell selection. The CDK1 lentivirus included pLenti-CMV-MCS-2A-mcherry-3FLAG-PGK-Zeo negative control vector (referred to as MSA), pLenti-CMV-CDK1-2A-mCherry-3FLAG-PGK-Zeo CDK1 overexpression lentivirus (referred to as CDK1) and pLenti-CMV-CDK1 (T14E, Y15D, T161A)-2A-mCherry-3FLAG-PGK-Zeo CDK1-MUTANT lentivirus (referred to as CDK1-MUT). We mutated at three sites (Thr-14, Tyr-15, and Thr-161) to inactivate CDK1 after searching Uniport dataset. When transduced, the cells were uniformly inoculated in a 6-well culture dish at a rate of 30%.

### Clinical tissues

Breast cancer tumor tissues and adjacent samples were used following approval from the ethical committee of the First Affiliated Hospital with Nanjing Medical University (Jiang Su Province Hospital). Samples remained in liquid nitrogen storage for further use once removed from the patients. Be sure that breast cancer tumor tissues and adjacent samples collection performed upon patients’ approval via written informed consent.

### CCK-8 assay

Based on the manufacturer’s instructions, we measured the proliferative capacity of breast cancer cells via the Cell Proliferation and Toxicity Test Kit (Dojindo, Japan).

### Colony formation assay

SUM-1315 and MCF-7 cell colony formation was assessed as in previous reports^20^.

### Tumor development in nude mice

Nude female BALB/C mice (4–6 weeks old, 18–22 g) were from Model Animal Research Center of Nanjing University (Nanjing, China). Animals were divided at random into two groups (*n* = 8 per group). SUM-1315 cells were stably overexpressed RBM7 or NC (1 × 10^6^ cells in a 100 µl PBS volume), followed by subcutaneous injection in the breast areas of nude mice. For recovery experiments, mice were divided into four groups, including SCR-MSA, Sh-1-MSA, Sh-1-CDK1, and Sh-1-CDK1-MUT. The growth of nude mice and tumors was investigated for 20 days. Every 4 days, tumor volumes were assessed using calipers based on the formula (tumor length × width^2^)/2. All samples were collected according to the ethics of Dec-Helsinki’s mourning and were approved by the First Affiliated Hospital Research Committee of Nanjing Medical University. The Institutional Animal Care and Use Committee of Nanjing Medical University approved these animal studies, which were consistent with the National Institutes of Health (NIH) care and use of Laboratory Animals guide.

### Dual luciferase reporter gene assay

Dual luciferase reporter assays conducted based on the provided directions and previously described techniques^22^. In brief, Cells were transfected in 24-well plates using both pGL3 reporter vectors as well as the control Renilla vector. Besides, B and C AREs mutant, called B-_MUT_ and C-_MUT_, generated the motif of ATTTTA in CDK1 3′-UTR into deletion mutation. Cells were then allowed to grow for 48 h before a Dual-Luciferase Reporter Assay System (E1910, Promega, WI, USA) was used for measuring luciferase activity.

### RNA immune-precipitation (RIP)

RIP was conducted as in previous reports by using the primary anti-rabbit RBM7 antibody (Sigma, USA)^44^. Protein A/G magnetic beads were employed for eluting immune-complexes. RT-PCR and RT-qPCR were utilized for measuring CDK1 mRNAs immune complexes in horizontal RBM7 or IgG. Add equal 10 μl to each well during the agarose gel electrophoresis experiment. Antibodies were diluted based on manufacturer’s recommendations.

### Immunohistochemistry (IHC) staining and analysis

TNM staging of clinical tissues was according to the American Joint Commission’s definition of cancer (AJCC) (6th edition, 2002). The antibody of RBM7 (Abcam, Cambridge, MA, USA) and CDK1 (Proteintech, USA) were used in the process of IHC staining.

### Cell cycle assay

Flow cytometry (BD, CA, USA) was used to assess cell cycle via collecting cells, washing them using PBS, fixing them at −20 °C in ethanol over an 8 h period, and then spinning them down. The cell cycle analysis of SUM-1315 and MCF-7 were conducted by Cell Cycle Staining Kit (Multi Sciences Biotech Co. Ltd) as previously described^22^.

### RNA sequencing analysis

Triplicate samples of 3 × 10^6^ well-conditioned RBM7 overexpression (RBM7) and control (NC) of MCF-7 cells were used for total RNA isolation. Transcriptome sequencing was performed by Beijing Allwegene Technology Co., Ltd. using Illumina Hiseq 4000 platform. The clean reads were mapped with the reference genome by Tophat2 tools soft (v2.1.0). Differentially expressed genes (DEGs) were those genes (FPKM > 1) with *P* < 0.05 found by DESeq (1.10.1). KOBAS was used for assessing KEGG pathway enrichment for these DEGs (Kyoto Encyclopedia of Genes and Genomes (http://www.kegg.jp). Our study didn’t involve in the content of ‘Guidance of the Ministry of Science and Technology (MOST) for the Review and Approval of Human Genetic Resources’.

### Cancer survival curve data analysis

Cancer survival curve data was obtained from The Human Protein Atlas (https://www.proteinatlas.org/)^45^.

### Statistical analysis

Experiments were conducted at least thrice unless otherwise specified. The *t*-test and one-way ANOVAs were conducted with SPSS 20.0 (IL, USA) to analyze the data sets, which were continuous variables. In vivo, the outliers were discarded due to the random errors when there was a big difference between groups. Correlations between RBM7 and patient clinicopathological findings were assessed via *X*^2^ test. Data are means ± standard deviation (SD). *P < 0.05 was the significance threshold.

### Reporting summary

Further information on research design is available in the Nature Research Reporting Summary linked to this article.

## Supplementary information

Supplementary Material

Reporting Summary Checklist

## Data Availability

The data generated and analysed during this study are described in the following data record: 10.6084/m9.figshare.12643508^46^. Datasets supporting Figs. 1–6, Supplementary figures 1 and 2 are publicly available in the figshare repository as part of the above data record^46^. The Cancer survival curve data are also publicly available in The Human Protein Atlas repository at: https://identifiers.org/hpa:ENSG00000076053^45^. RNA sequencing data of RBM7 on global RBM7 gene regulation are publicly available in the NCBI Sequence Read Archive (SRA) repository at: https://identifiers.org/ncbi/insdc.sra:SRP274505^47^. Full and uncropped Western blots are available as part of the supplementary information (Supplementary Fig. 4).
