# Supplementary material for: Oncogenic action of the exosome cofactor RBM7 by stabilization of CDK1 mRNA in breast cancer
Source: NPJ Breast Cancer. 2020 Oct 30;6:58. doi: 10.1038/s41523-020-00200-w (PMC7603334; doi:10.1038/s41523-020-00200-w)

Supplementary Material

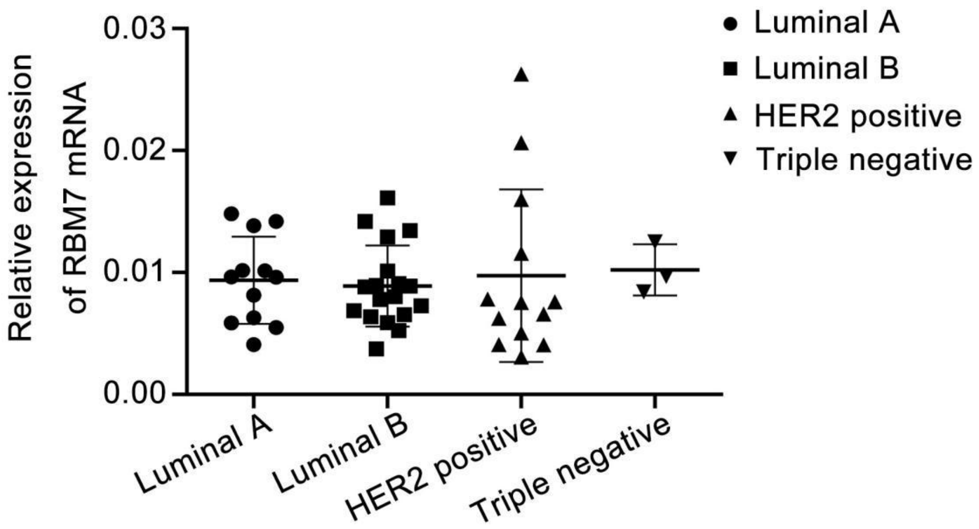

Supplementary Figure 1: Expression of RBM7 in different breast cancer molecular subtypes.

The expression level of RBM7 mRNA in human breast cancer tissues according to four different breast cancer molecular subtypes (Luminal A, Luminal B, HER2 positive and Triple negative). The relative quantification was calculated by the $2^{-\Delta Ct}$ method and normalized based on β-actin (ns, P>0.05).

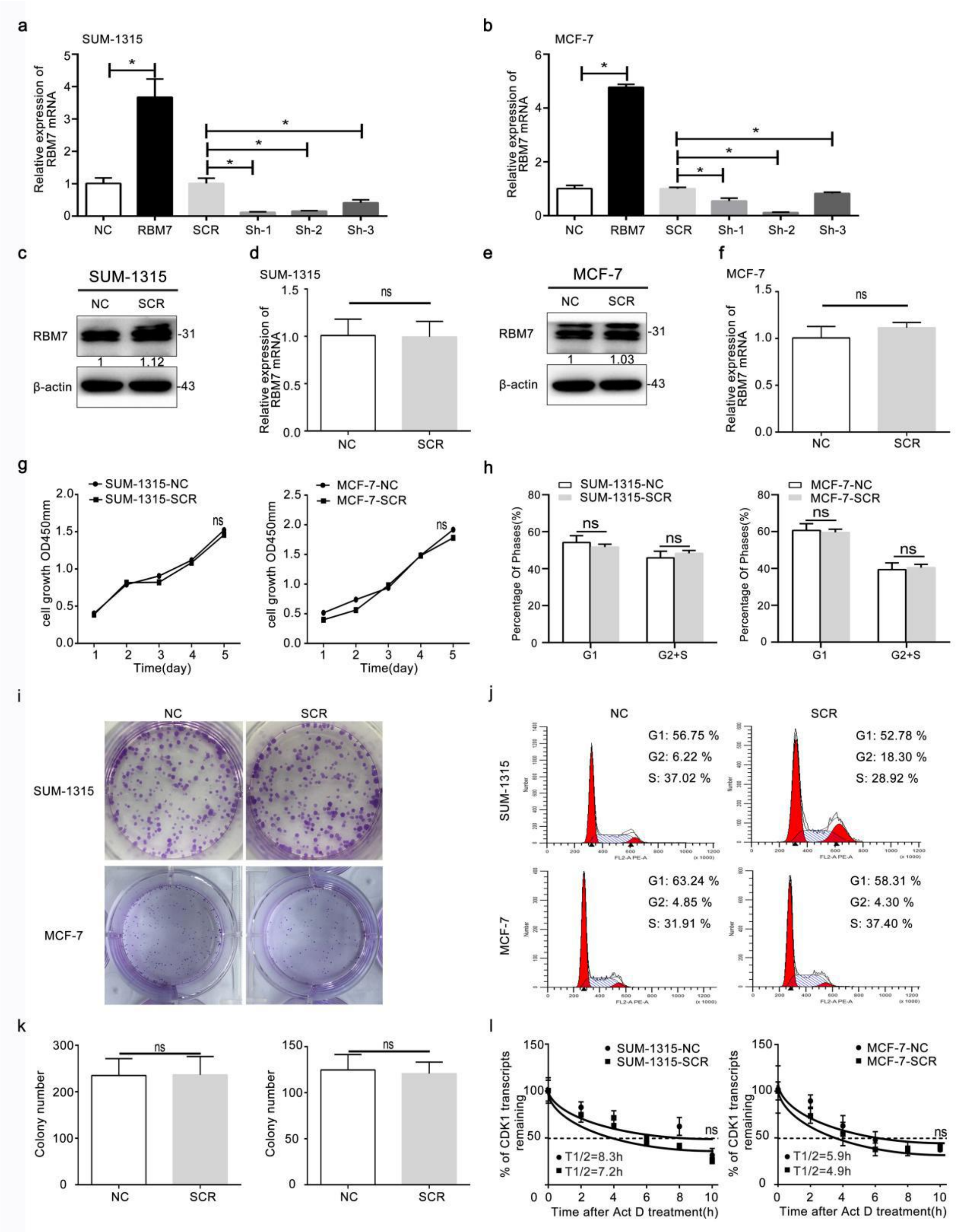

Supplementary Figure 2: Comparison of NC and SCR group in different experiments.

RT-qPCR analysis of RBM7 mRNA expression in SUM-1315 (**a**) and MCF-7 (**b**) cells. The relative quantification was calculated by the $2^{-\Delta\Delta Ct}$ method and normalized based on β-actin (*P<0.05). Western blot was used to demonstrate the efficiency of

NC and SCR in SUM-1315 (**c**) and MCF-7 (**e**) cells. A fold change in the RBM7 protein was shown below each lane. The intensity of the bands was determined using densitometric analysis. RT-qPCR was used to verify the efficiency of NC and SCR in mRNA level in SUM-1315 (**d**) and MCF-7 (**f**) cells. The relative quantification was calculated by the $2^{-\Delta\Delta Ct}$ method and normalized based on β-actin. The data were represented as mean ±SD (ns, P>0.05). (**g**) The growth of SUM-1315 and MCF-7 cells of NC and SCR over 5 days was measured using CCK-8 assays. The data were represented as mean ± SD (ns, P>0.05). (**h** and **j**) Cell cycle progression of NC and SCR was measured using flow cytometry in SUM-1315 and MCF-7 cells. The data were represented as mean ± SD (ns, P>0.05). (**i** and **k**) The growth of cells over 14 days of NC and SCR was measured using colony formation assays. Columns: average of three independent experiments (ns, P>0.05). Colonies>50 mm were counted. (**l**) The half-life of CDK1 transcript was measured in SUM-1315 and MCF-7 cells. The NC and SCR cells were both treated with 5 μg/ml Act D for 0, 2, 4, 6, 8, 10 h. The relative quantification was calculated by the $2^{-\Delta\Delta Ct}$ method and normalized based on β-actin (ns, P>0.05).

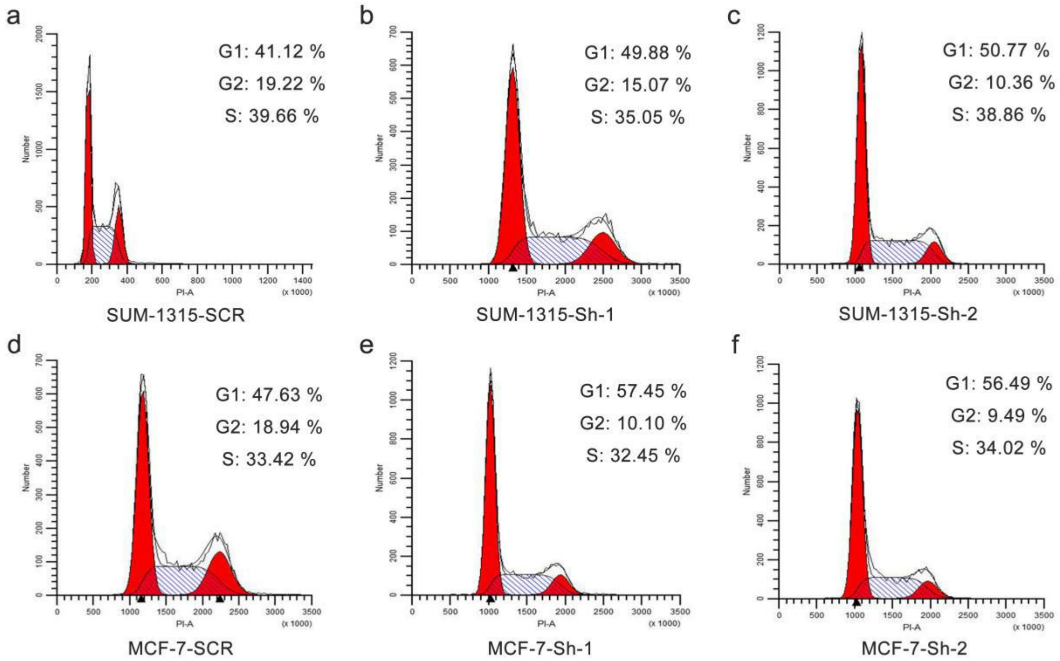

Supplementary Figure 3: Knockdown of RBM7 inhibited breast cancer cell cycle progression.

Cell cycle progression was measured by using flow cytometry after knockdown of RBM7 in SUM-1315 (**a**, **b** and **c**) and MCF-7 cells (**d**, **e** and **f**). The data were represented as mean ± SD (**P*<0.05).

Supplementary Figure 4：(Raw data for the blots included in each figure and supplementary figure.)

Figure 1b

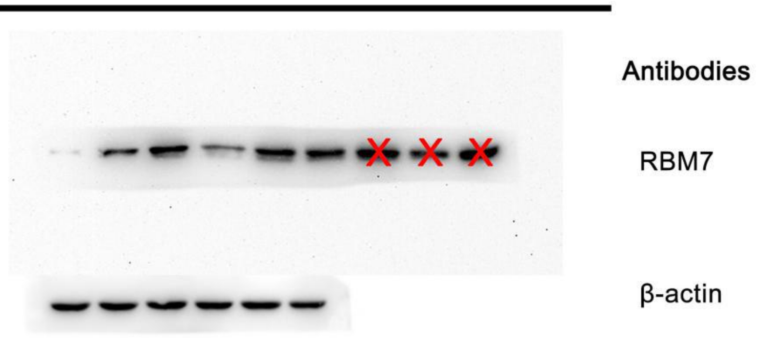

Figure 1d

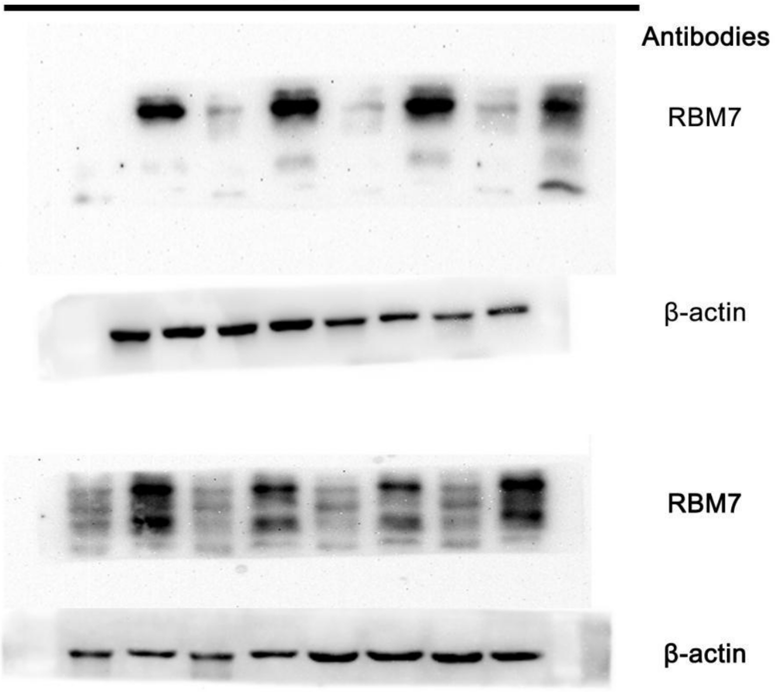

Figure 2a

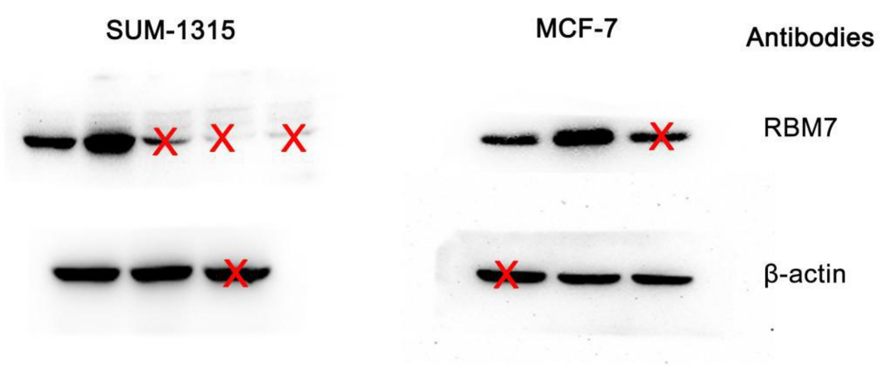

Figure 2c

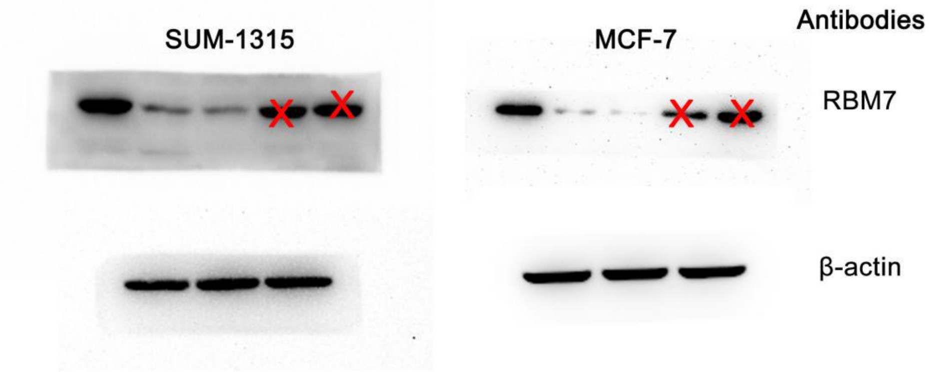

Figure 4e

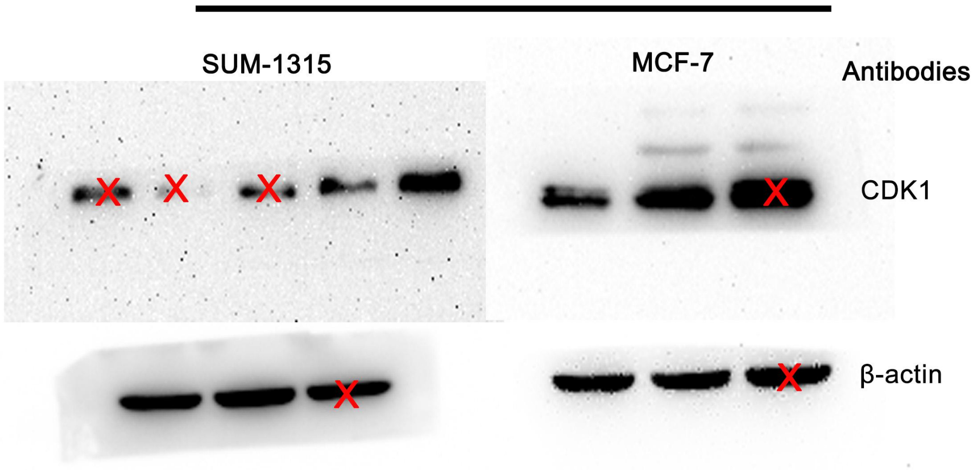

Figure 4g

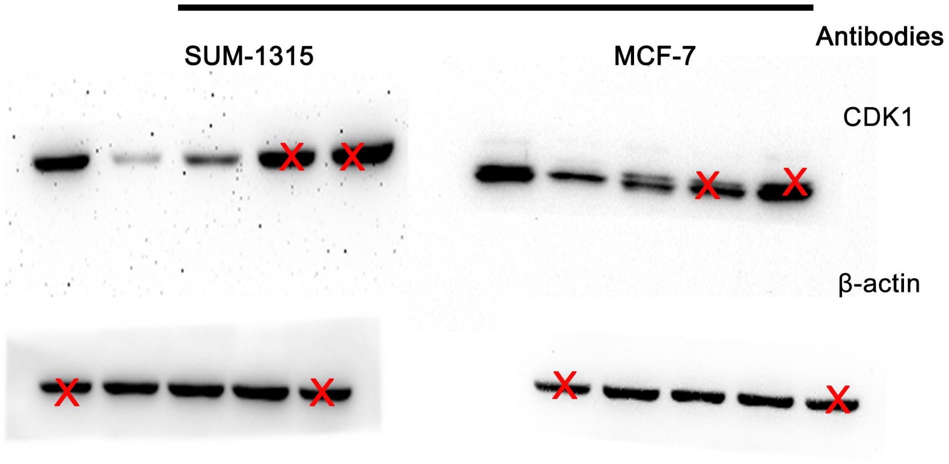

Figure 5e

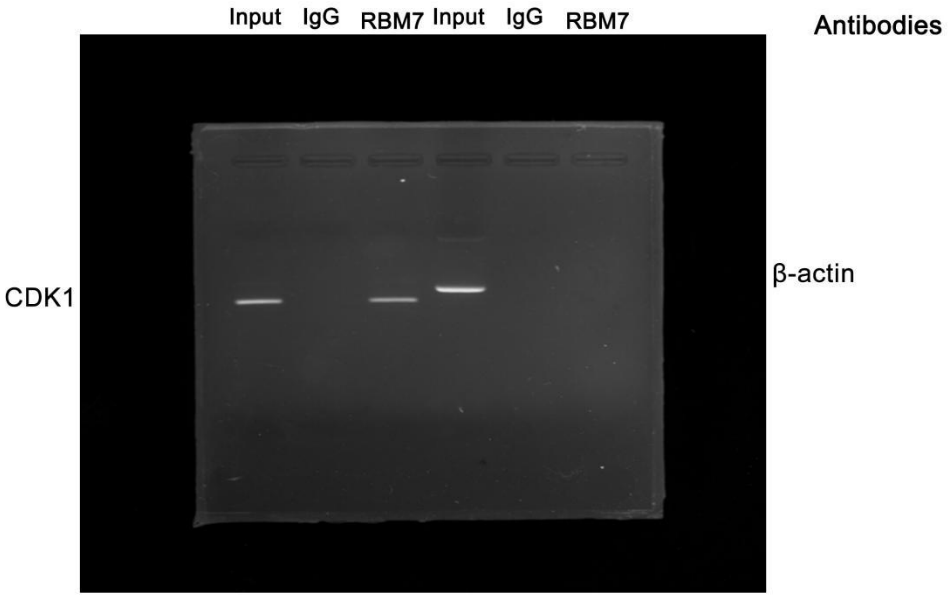

Figure 5g

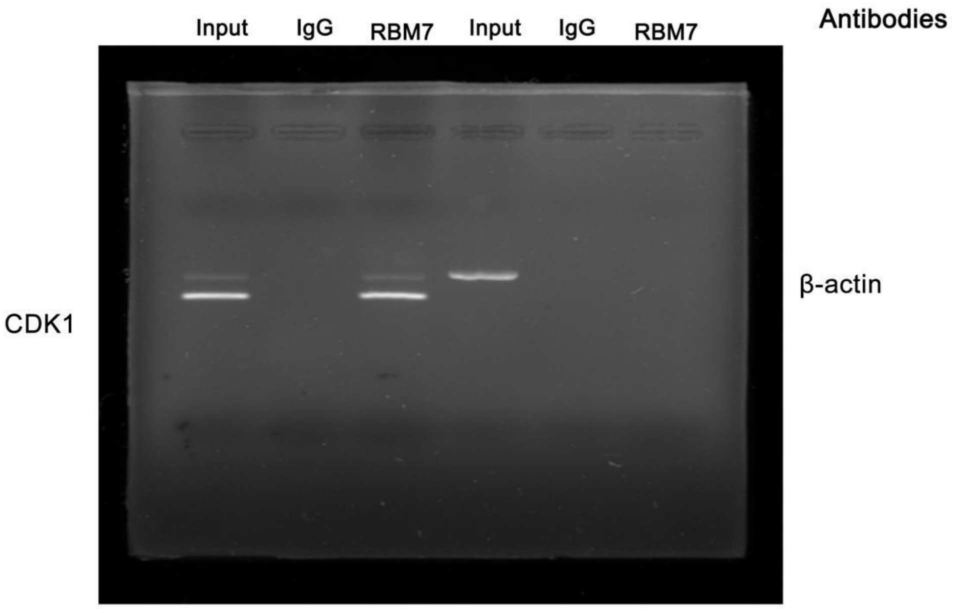

Figure 6a

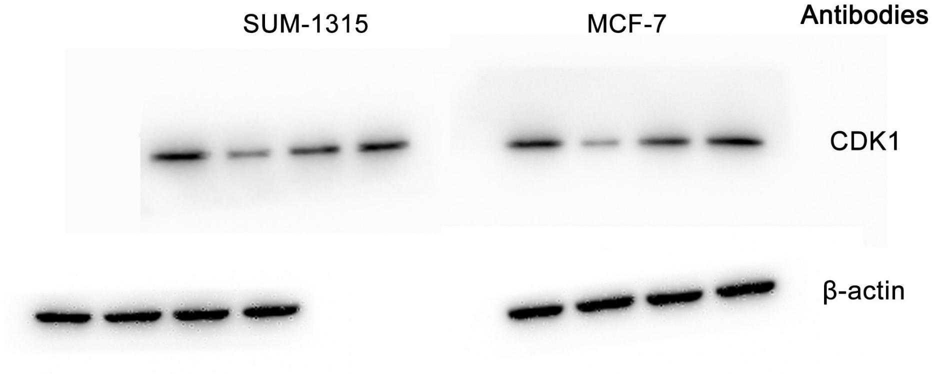

Figure 6k

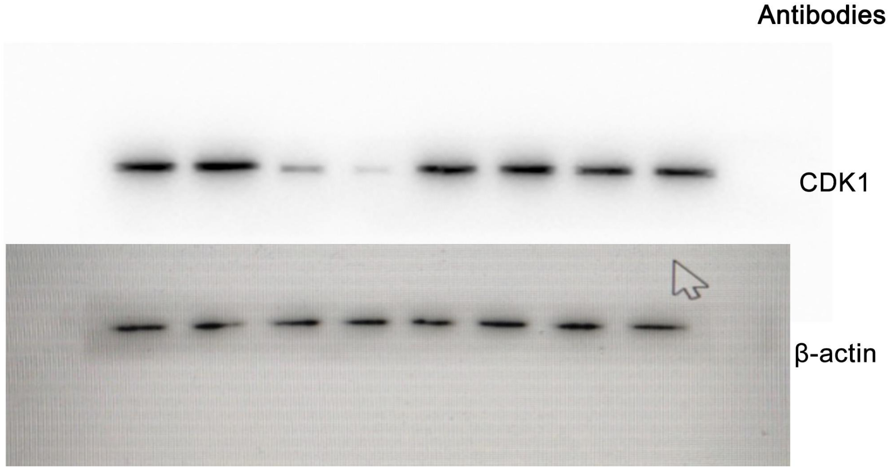

Supplementary Figure 2c

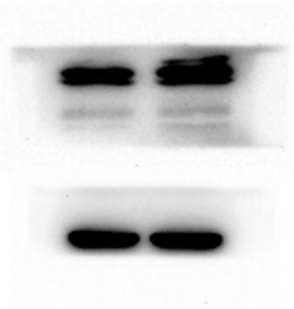

Supplementary Figure 2e

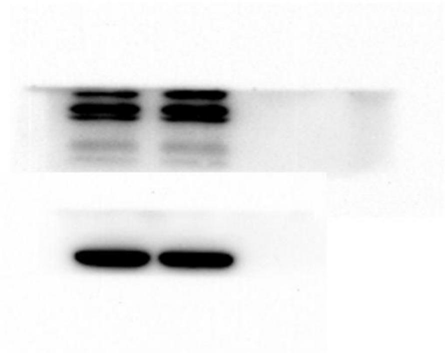

Supplement: Supplementary file 1 — Supplementary Material [file 41523_2020_200_MOESM1_ESM.pdf]
